# Participant demographics reported in "Table 1" of randomised controlled trials: a case of "inverse evidence"?

**DOI:** 10.1186/1475-9276-11-14

**Published:** 2012-03-19

**Authors:** John Furler, Parker Magin, Marie Pirotta, Mieke van Driel

**Affiliations:** 1 Department of General Practice, University of Melbourne, 200 Berkeley St, Carlton, VIC 3053, Australia; 2 Discipline of General Practice, Newbolds Bldg, University of Newcastle, University Dr, Callaghan 2308, Australia; 3 Department of General Practice, University of Melbourne, 200 Berkeley St, Carlton, VIC 3053, Australia; 4 Discipline of General Practice, University of Queensland, Brisbane 4029, QLD, Australia; 5 Department of General Practice and Primary Health Care, Ghent University, Ghent, Belgium

## Abstract

**Introduction:**

Data supporting external validity of trial results allows clinicians to assess the applicability of a study's findings to their practice population. Socio-economic status (SES) of trial participants may be critical to external validity given the relationship between social and economic circumstances and health. We explored how this is documented in reports of RCTs in four major general medical journals.

**Methods:**

The contents lists of four leading general medical journals were hand searched to identify 25 consecutive papers reporting RCT results in each journal (n = 100). Data on demographic characteristics were extracted from each paper's Table 1 only (or equivalent).

**Results:**

Authors infrequently reported key demographic characteristics relating to SES of RCT participants. Age and gender of participants were commonly reported. Less than 10% reported occupational group, employment status, income or area based measures of disadvantage.

**Conclusions:**

Without adequate reporting of key indicators of SES in trial participants it is unclear if lower SES groups are under-represented. If such groups are systematically under-recruited into trials, this may limit the external validity and applicability of study findings to these groups. This is in spite of the higher health-care need in more disadvantaged populations. Under-representation of low SES groups could underestimate the reported effect of an intervention for those with a higher baseline risk. The marginal benefit identified in a trial with poor or no representation of lower SES participants could significantly underestimate the potential benefit to a low SES community. More transparency in this reporting and greater attention to the impact of SES on intervention outcomes in clinical trials is needed. This could be considered in the next revision of the CONSORT statement.

## Introduction

The CONSORT statement [1] encourages authors reporting randomised controlled trial (RCT) results to include "baseline demographic and clinical characteristics for each group" of participants. This baseline information should be presented in a table, effectively becoming the "Table 1" in published reports of RCTs.

Demographic, from "demography", refers to characteristics related to the "life-conditions of communities of people" [2]. Significantly, the CONSORT statement is not prescriptive as to which demographic features should be reported, giving no indication as to which are important or useful to readers.

CONSORT notes that one reason for reporting this data is to assess comparability of study groups. Study groups "should be compared at baseline for important demographic and clinical characteristics so that readers can assess how comparable the groups were".

Another reason for reporting of baseline demographic characteristics is for assessment of external validity of trial results. This is of relevance to clinicians who must assess the applicability of study findings to their practice populations and to individual patients particularly in primary care dealing with communities and people with multiple conditions and vulnerabilities [3]. For example, age and sex of trial participants is of obvious relevance to external validity of a trial. A good illustration of this is the relative lack of representation of women and the elderly in trials of interventions in ischaemic heart disease which makes applicability of their results to those patient groups less certain [4].

Another critical demographic characteristic of study populations that may impact upon assessment of external validity is socio-economic status (SES). SES of a study participant may be inferred from individual characteristics such as employment status, occupation, educational achievement or place of residence (where an area based measure of socioeconomic disadvantage is available such as the Socio-Economic Indexes for Areas, Index of Relative Socio-Economic Disadvantage (SEIFA IRSD) in Australia for example [5]). There is now overwhelming evidence of the relationship between social and economic circumstances and health and inequities in health [6]. There are many ways that SES may affect the external validity of a trial's results. For example, if an intervention is trialled in a population of high SES patients, co-morbidity is likely to be less than in lower SES groups: how applicable these results would then be to a disadvantaged population where co-morbidity is a key consideration is unclear. Similarly, theoretically an educational intervention that requires a high level of literacy trialled in a relatively well-educated study population may be less applicable to a practice population where education and literacy levels are lower. While re-analysis of observational studies on hormone replacement therapy in postmenopausal women revealed the pivotal role of SES as a confounder [7], sub-analyses from interventional studies are uncommon.

The aim of this paper is to identify the extent to which demographic attributes, in particular the SES characteristics, of trial participants are documented in the reporting of randomised controlled trials in four major general medical journals.

## Methods

The contents lists of four leading international general medical journals, the New England Journal of Medicine, the Journal of the American Medical Association, the Lancet and the British Medical Journal, were hand searched to identify papers reporting RCT results. Working backwards in time from February 1^st ^2010, 25 consecutive papers reporting RCT results were identified in each of the four journals (N = 100).

Inclusion criteria for papers were: reporting a randomised controlled trial; a clinical trial in human subjects; data collection and analysis at the level of the individual participant; phase 3 or 4 trials; and reporting of a single trial. As the focus of this analysis was on generalizability we included published RCTs covering a wide range of conditions, populations and interventions.

Data on the reporting of demographic characteristics were extracted from each paper's Table 1 (or equivalent). Data extraction was limited to data on characteristics of patients included in the studies as appearing in tabular form and as available to readers, as this is the CONSORT recommendation. The sampling frame or methods of each trial were not the focus of this analysis.

The choice of most relevant demographic factors to be included was made by author consensus. Included for data extraction were: age, sex, marital status, ethnicity, language use, geographic or area-based measures (eg rural/urban location, area measure of disadvantage, distance from the trial centre), employment status, occupational status, income, and educational attainment. In studies with children we assessed reported data for children as well as parents. If any of the variables for either of the groups was mentioned in the table they were classified as "reported". We also extracted data on whether other demographic markers of life conditions were reported including weight/BMI, smoking and alcohol use. We regarded gender as reported if a trial was gender specific. A data extraction form was constructed which included dichotomous responses for each of the identified demographic factors (reported/not reported).

Each of the four authors extracted data from two journals (BMJ/Lancet or NEJM/JAMA) resulting in two independent assessments of whether demographic data were reported in each paper. Differences in assessment of data from individual papers were resolved by consensus.

Results are presented as percentage of eligible papers reporting each of the demographic features of their study populations.

## Results

Results are presented in the Table 1, which shows the percentage of papers reporting each included socio-demographic variable. Twelve papers reported on RCTs in gender specific populations and nine in children and infants.

**Table 1:** Reporting of demographic variables in 100 RCTs published in four leading general medicine journals

Demographic variable	Percentage of papers reporting that variable
Age	99

Sex	99

Weight/BMI	39

Ethnicity	37

Smoking	24

Educational attainment	23

Marital Status	13

Any area-based measure (eg index of deprivation or disadvantage, rurality distance from health centres etc)	10

Employment status	10

Alcohol	4

Occupational status	2

Income	2

Language spoken	0

## Discussion

We found that reports of RCTs poorly documented key demographic characteristics relating to SES. Almost all trials reported age and gender of participants. Ethnicity and education level were the next most commonly reported variables. Very few trials reported occupational group, employment status, income or area based measures of disadvantage.

The relevance of any one or more demographic characteristics to the internal and external validity of any individual trial will vary depending on the intervention being trialled. Nevertheless, while CONSORT is not prescriptive as to which baseline demographic variables should be reported, the intent of the statement is to "facilitate clarity, completeness, and transparency of reporting" noting that "explicit descriptions, not ambiguity or omission, best serve the interests of all readers". It seems unlikely that the proportions of papers found to be reporting detailed baseline characteristics in this study are consistent with this stated intent. Clinicians, particularly primary care clinicians, seeking to determine the applicability of trial results to their patients may not be provided with sufficient data to do so.

Of particular note is our finding of the relative lack of reporting of SES of trial participants. This makes it impossible to assess whether low SES groups are adequately represented in trial populations. As noted earlier, there is a range of ways that SES may be important to assessing a trials external validity. It has been suggested that lower SES groups may be under-represented in trials [8,9] but without adequate reporting of this key characteristic this is unclear.

There are good reasons why people from more disadvantaged circumstances may be under-represented in trials. These groups have difficulties in accessing health care so may also be likely to have difficulty with "accessing" trials. For example, complying with private transport or communication technology requirements of study protocols may be a barrier. Lower levels of literacy or speaking a language other than that used in study documents may tend to exclude people from some trials. Co-morbidity, more common in more disadvantaged populations [10] is also likely to lead to exclusion from trials with strict inclusion criteria. If subjects from lower SES background *are *systematically under-recruited into trials, the external validity of study findings to these groups may be limited. Higher health-care need in more disadvantaged populations is well documented. Lower SES patients have higher prevalence of disease, more multi-morbidity, more psychological co-morbidity and illness has a greater impact on their lives [10]. In addition to the recognised "inverse care law" [11], our results identify a potential "inverse evidence law" which implies limitations in generalisability of trial findings for lower SES groups.

An intervention that has an effect in a trial may have little effect in people of low socio-economic status for many reasons, for example, they may receive many drugs already and they may have low compliance with medicine or whatever treatment has been investigated. They may also be so ill because of co-morbid diseases that the effect would not have been reproduced among them, if a trial had been undertaken which included adequate representation from such groups.

The opposite possibility is equally important. If the under-reporting of SES does in fact reflect under-representation of low SES groups, this may also mean that the effect of an intervention could be underestimated for the very group for whom it is likely to offer the greatest benefit. An intervention might be found to have a marginally significant effect in an RCT with an under-representation of lower SES subjects, where trial participants have an overall lower burden of disease. The marginal benefit identified in such an unrepresentative trial could significantly underestimate the potential benefit to a community starting with higher need and higher baseline risk.

In this study we illustrate how SES characteristics are reported. There may be many reasons for study authors not to report this in Table 1 even though they have collected the data. However, the CONSORT statement is designed to assist systematic assessment of generalizability of results. Time-poor clinicians therefore might rely on Table 1 to assess the relevance of the study findings to their patients.

While our analysis is based on a convenience sample of medical journals reporting RCTs, the papers were drawn from leading general medical journals and if anything we would expect this to lead to a bias towards more complete reporting. Future studies should examine the reporting of sampling frames and could focus particularly on trials in areas where socio-economic background is known to have an impact on the outcome.

## Conclusions

Reporting of socio-economic characteristics of clinical trial participants is limited. This reduces our ability to assess the generalisability of study findings to day-to-day clinical practice, particularly in primary care. Moreover, if hidden behind this lack of reporting is a systematic exclusion of lower SES participants from clinical trials, this might underestimate potential health gains, and in absence of this information we might be dismissing interventions that could benefit disadvantaged populations and help to reduce health inequity. More transparency in this reporting and greater attention to the impact of SES on intervention outcomes in clinical trials is needed. It may be important for the next revision of the CONSORT statement to consider explicit reporting of relevant SES as one of its criteria.

## Competing interests

The authors declare that they have no competing interests.

## Authors' contributions

All authors contributed to the development and writing of the paper. JF is the guarantor of the paper and analyses. All authors read and approved the final manuscript.
